# Assessment of resilient modulus of soil using hybrid extreme gradient boosting models

**DOI:** 10.1038/s41598-024-81311-3

**Published:** 2024-12-30

**Authors:** Xiangfeng Duan

**Affiliations:** Imperial College London, London, UK

**Keywords:** Soil resilient modulus, Regression prediction, Black-winged kite algorithm, Extreme gradient boosting, Computer science, Civil engineering

## Abstract

Accurate estimation of the soil resilient modulus (M_R_) is essential for designing and monitoring pavements. However, experimental methods tend to be time-consuming and costly; regression equations and constitutive models usually have limited applications, while the predictive accuracy of some machine learning studies still has room for improvement. To forecast M_R_ efficiently and accurately, a new model named black-winged kite algorithm-extreme gradient boosting (BKA-XGBOOST) is proposed. In BKA-XGBOOST, XGBOOST captures the many-to-one nonlinear relationship between geotechnical factors and M_R_, while BKA provides the optimal hyperparameters for XGBOOST. By combining them, XGBOOST has stable and accurate predictive capabilities for different combinations of soil data. Comparisons with nine models show that the proposed model outperforms other models in terms of M_R_ prediction accuracy, with a determination coefficient (R^2^) of 0.995 and a mean absolute error (MAE) of 0.975 MPa. In addition, an efficient M_R_ prediction software is developed based on the model to improve its practicality and interactivity, which is promising for assisting engineers in evaluating pavement properties.

## Introduction

The advent of modern transportation systems has led to an increase in the construction of roads. The elastic modulus of recoverable strain under repeated stress is called resilient modulus (M_R_). It is defined as the ratio of the deviator stress (σ_d_) to the recoverable strain (ε_r_). As one of the essential characteristics of soil, M_R_ has been found to depend on several factors such as the weighted plasticity index (wPI), dry unit weight (γ_d_), confining stress (σ_c_), deviator stress (σ_d_), moisture content (w) and the number of FT cycles (N_FT_)^1–4^. The prediction of M_R_ is of paramount importance to the safe and sustainable design of flexible pavement systems^5,6^. Traditional forecasting methods for M_R_ can be classified into three primary categories: experimental methods, regression equation methods, and principal modeling methods (as illustrated in Table 1). Owing to their complexity and time consumption, these technologies are challenging to implement in engineering.

**Table 1:** The traditional methods for predicting M_R_.

Methods	Examples	Shortcomings
Experimental methods	Cyclic triaxial load testing, torsional shear testing and resonant column testing^7–13^	Expensive, time-consuming and complex^6,14^
Regression equation methods	$\:\frac{{M}_{R}}{{P}_{a}}={k}_{1}{\left[\frac{{P}_{a}\cdot\:{\sigma\:}_{oct}}{{\tau\:}_{oct}^{2}}\right]}^{{k}_{2}}\:,\:\:{M}_{R}={k}_{1}{P}_{a}{\left[\frac{9{P}_{a}}{2}\left(\frac{1}{3{\sigma\:}_{d}}+\frac{{\sigma\:}_{s}}{{\sigma\:}_{d}^{2}}\right)\right]}^{{k}_{2}}$ ^15^ $\:{M}_{R}={k}_{1}{P}_{a}{\left(\frac{\theta\:}{{P}_{a}}\right)}^{{k}_{2}}{\left(\frac{{\tau\:}_{\text{ott\:}}}{{P}_{a}}+1\right)}^{{k}_{3}}\:$ ^16^ $\:{M}_{R}={k}_{1}{\sigma\:}_{3}^{{k}_{2}}{\sigma\:}_{d}^{{k}_{3}}$ ^17^	Complex and limited scope of application^18^
Constitutive model methods	*k-θ* model ^19^, deviatoric stress model^20^, Uzan model^21^, general model^22^, suction-based model^23–26^, moisture content-based model^27,28^, saturation-based models^29^, dry density- based models^30–32^, plasticity index-related models^8,33,34^	Complex, time-consuming and limited scope of application^35^

On the other hand, developing intelligent computing technologies, including support vector machines, long-short-term neural networks, random forests, and artificial neural networks, has enabled the rapid, effective, and cost-effective resolution of some civil engineering problems^36–45^. As illustrated in Table 2, some researchers have employed various machine learning techniques to predict the M_R_, either by utilizing single or combinatorial algorithms. These studies demonstrate that machine learning methods are proficient at predicting M_R_.

**Table 2:** Application of machine learning for predicting M_R_.

Algorithms	
Single algorithm	Artificial neural network^14,46–52^
	Extreme learning machine^53^
	K-nearest neighbor^54,55^
	Support vector machine^56^
	Extreme gradient boosting^57^
	Random forest^51,57,58^
	Least square support vector machine^59^
Combinatorial algorithms	Particle swarm optimization- extreme gradient boosting^60^
	Genetic algorithm-adaptive layered population structure^61^
	Symbiotic organisms search-least square support vector machine ^62^
	Jellyfish swarm optimizer- extreme gradient boosting^63^
	Colliding bodies optimization- support vector machine^6^
	Genetic algorithm-artificial neural network^64,65^

### Research gap

However, there is room for improvement in some of these machine learning studies. The accuracy of the models presented in the article can be improved. Fewer soil types were used in the experiments, making it difficult to demonstrate the generalizability of the models. Moreover, non-computer individuals have difficulties in using the models. Therefore, there is a need for more research in machine learning for predicting M_R_.

### Research objective

This paper aims to develop an efficient, generalizable and user-friendly M_R_ prediction model. For that purpose, this study proposed the black-winged kite algorithm-extreme gradient boosting model (BKA-XGBOOST) and collected 12 types of soil data from multiple studies to verify the feasibility and generalizability of the model. The model was based on XGBOOST, well-suited for capturing many-to-one nonlinear relationships^66–70^. Moreover, BKA, with powerful optimization capabilities^71^, was employed to optimize the parameters of XGBOOST, ensuring that XGBOOST maintains optimal performance. In addition, an application program based on BKA-XGBOOST has been developed to facilitate its use by engineers.

### Research significance

This study addresses the problem of M_R_ being difficult to assess in the geotechnical field. Traditional methods for predicting M_R_ are complex and time-consuming, and some machine learning models exhibit low accuracy and inadequate generalizability. By proposing the BKA-XGBOOST model, this study introduces a novel approach to accurately and efficiently assess various types of M_R_. The application developed based on the proposed model is user-friendly and can assist engineers in geotechnical engineering.

## Research methodology

**Fig. 1:**
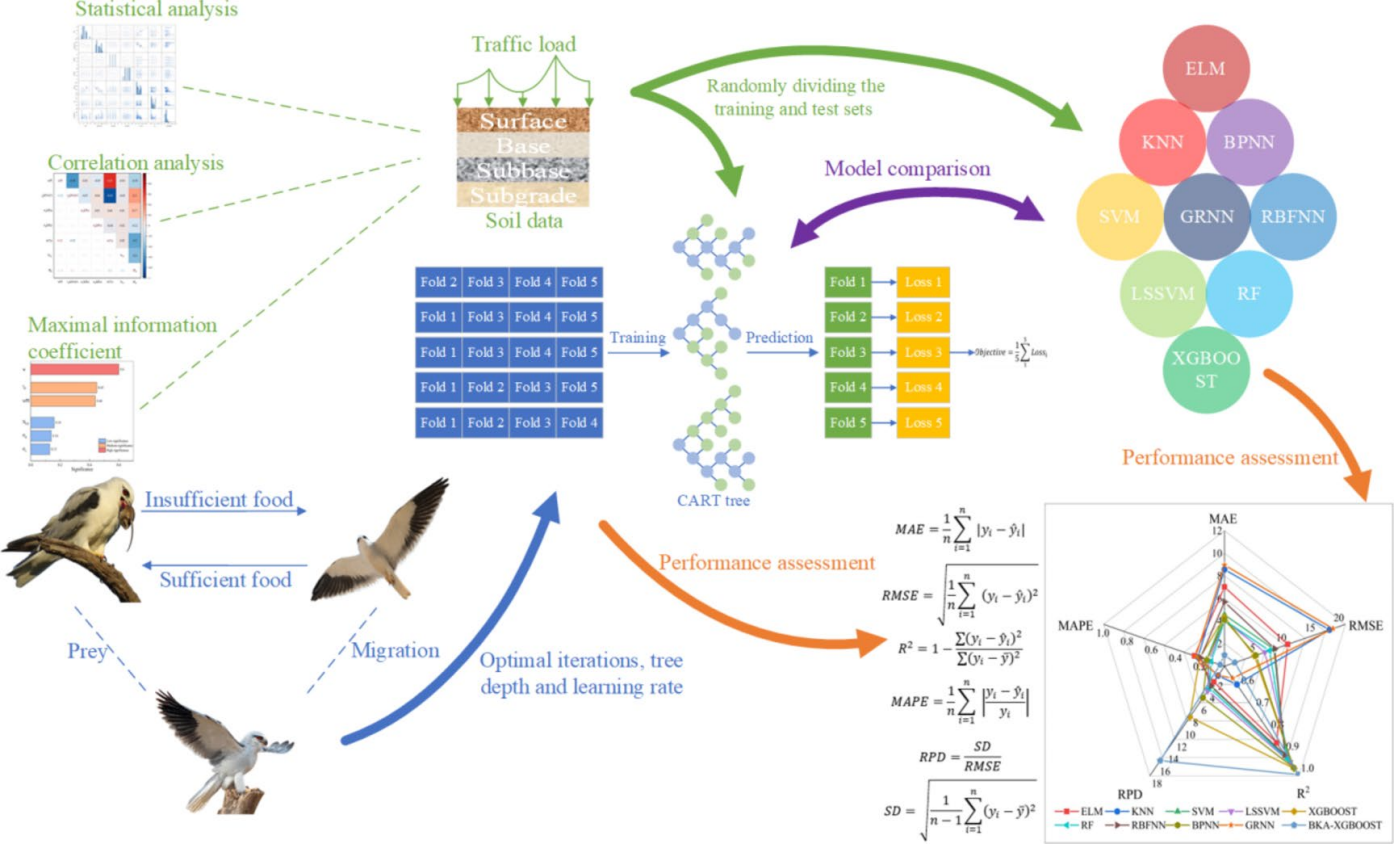
The structure of the research methodology.

The BKA-XGBOOST model proposed in this paper employs BKA to search for the three best parameters of XGBOOST to improve its prediction accuracy for various soil data. The dataset utilized for the experiment was collected from Ding, Rahman, Solanki and Ren^2–4,72^, encompassing 2813 soil data points. To evaluate the performance of the proposed model, it was compared with nine single models and four hybrid models utilized in previous studies from a range of evaluation metrics. In addition, a BKA-XGBOOST-based application was developed to improve the model’s usability. Figure 1 illustrates the structure of the research methodology for this study.

### Experimental environment

The training and testing were conducted using a hardware setup that consists of a 12th Gen Intel (R) Core i5-12500 H processor, 32.0 GB of RAM, and an NVIDIA GeForce RTX 3060 graphics card. The utilized software is MATLAB 2023b. Furthermore, the proposed model requires an average of 0.5247 s to predict 563 samples within the specific hardware setting.

## Data analysis

**Fig. 2:**
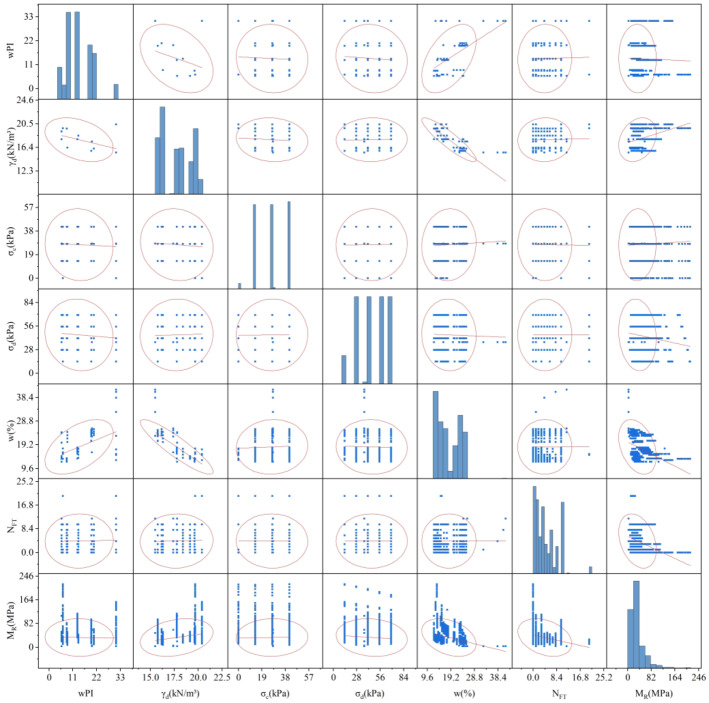
Soil data statistics.

Several factors affect M_R_. Choosing the wrong factors as inputs to XGBOOST would lead to imprecise forecasts and unnecessary computations. Some researchers have demonstrated that the wPI, γ_d_, σ_c_, σ_d_, w and N_FT_ exert a more pronounced influence on M_R_^1–4^ ; hence, this study employs these factors as inputs. To validate the feasibility of the proposed model, this study gathered 2,813 sets of soil data from earlier studies as experimental data^2–4,72^. The dataset comprises six inputs and one output. wPI equals the plasticity index measured by the standard plasticity test multiplied by the percent passing 0.425 mm sieve; γ_d_ is the dry weight of the soil divided by the soil’s volume; σ_c_, σ_d_ and M_R_ are obtained from the triaxial test; w is typically calculated by determining the mass difference after drying the soil; N_FT_ is derived from cyclic freeze-thaw experiments. Twelve soil types were included in the dataset, with two originating from China, five from the United States, and five from Canada. According to the statistical analysis in Table 3, it can be seen that the value distribution of γ_d_ is relatively concentrated, while the distribution of other inputs is relatively scattered. In particular, a larger standard deviation for σ_c_, σ_d_, and M_R_ implies more significant fluctuations in their values. From Fig. 2, all the variables soil data variables exhibit a non-positive distribution, and the relationship between the two variables is complicated, encompassing negative, positive, and weak correlations. For instance, the sub-graphs in the first column of the fifth row and the second column of the fifth row illustrate that w tends to increase with wPI, contrary to γ_d_. On the other hand, σ_d_ remains constant as σ_c_ escalates. It is essential to analyze the relationship between the variables further.

**Table 3:** Soil data statistics.

	wPI	γ_d_ (KN/m^3^)	σ_c_ (kPa)	σ_d_ (kPa)	w (%)	*N*_FT_	M_*R*_ (MPa)
Minimum	5.82	15.50	0.00	13.80	12.30	0.00	3.00
Maximum	31.08	20.40	41.40	68.90	41.54	20.00	217.00
Average	13.88	17.73	27.17	45.64	18.36	4.13	33.81
Standard error of mean	0.12	0.03	0.22	0.33	0.09	0.07	0.50
Standard deviation	6.43	1.56	11.85	17.34	4.52	3.93	26.62

**Fig. 3:**
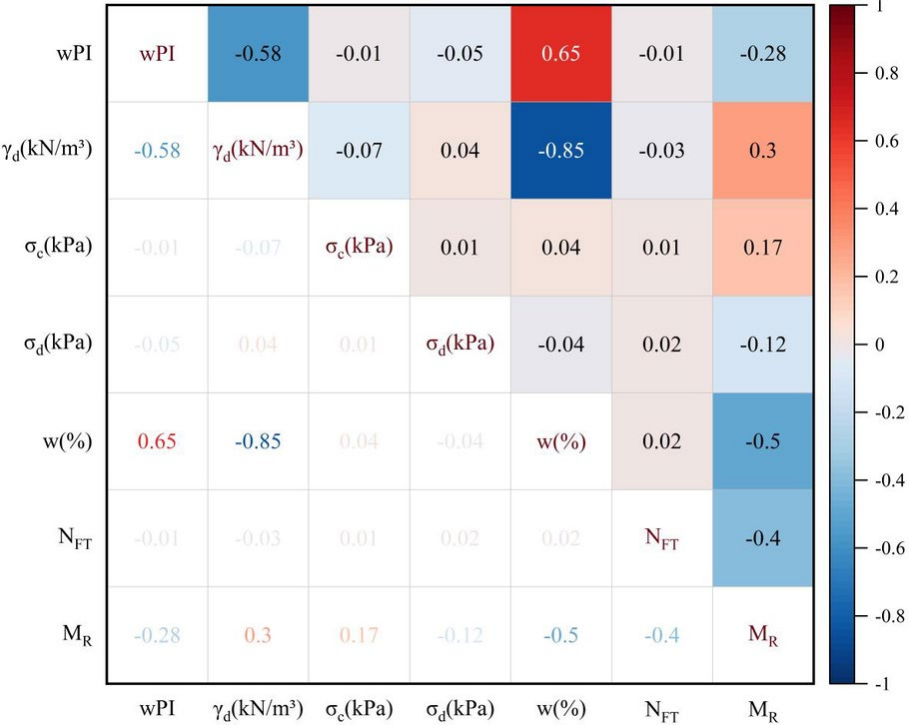
Correlations between variables.

Correlation analysis was employed to investigate the relationship between these variables further. Pearson, Spearman, and Kendall are three standard methods for analyzing the correlations between two variables. The Pearson method is suitable for normally distributed continuous variables. In contrast, the Spearman method is suitable for non-normally distributed continuous variables, and the Kendall method is suitable for categorical variables. Because all variables in the data were non-normally distributed, the Spearman correlation coefficient was employed^73^. If the correlation coefficients between two variables range from ± 0.81 to ± 1.0, ± 0.61 to ± 0.80, ± 0.41 to ± 0.60, ± 0.21 to ± 0.40, and ± 0.00 to ± 0.20, respectively, then they may show a very strong, strong, moderate, weak, and no relationship^74^. From Fig. 3, it shows that γ_d_ and w present a very strong negative correlation, wPI and w exhibit a strong positive correlation, while wPI and γ_d_, as well as w and M_R_, show moderate negative correlations. The remaining variables display either weak correlation or no correlation with one another. Although γ_d_ and w, as well as wPI and w, exhibit a strong correlation, the rest of the variables have a weak correlation. Consequently, it was unnecessary to assess the data for multicollinearity.

## Soft computing approaches

### Extreme gradient boosting

XGBOOST, the foundation of the proposed model, was initially introduced by Chen in 2016^75^. It represents an improvement upon the gradient-boosting decision tree (GBDT). Compared to GBDT, XGBOOST exhibits the following principal advantages: (1) The CART tree produced by XGBOOST takes into account the intricacy of the tree. (2) XGBOOST is a second-order derivative expansion that fits the previous round of loss function. In contrast, GDBT is a first-order derivative expansion that fits the previous round of loss function. Hence, XGBOOST exhibits higher accuracy and necessitates a reduced number of iterations. (3) XGBOOST can utilize multi-threading throughout the process of selecting the optimal split point, resulting in a significant enhancement in the speed of execution. (4) XGBOOST integrates regularization terms into the loss function, which helps control the complexity of the model and prevents overfitting. (5) XGBOOST permits the utilization of custom objective functions and evaluation functions, provided that the objective function is second-order differentiable^76^. (6) XGBOOST can automatically learn the trend of missing feature values in sample^77^. Regarding regression problems, the fundamental algorithm of XGBOOST mainly involves determining expected M_R_ and objective functions. The specific contents of this algorithm are as follows:

For soil data $\:\left\{\left({x}_{1},{\text{y}}_{1}\right),\dots\:,\left({x}_{n},{y}_{n}\right)\right\}$, containing n samples, the predicted value of M_R_ can be expressed as:

1 $$\:\begin{array}{c}{\hat{y}}_{i}=\sum\limits_{k=1}^{K}\:{f}_{k}\left({x}_{i}\right)\:{f}_{k}\in\:F,\end{array}$$

where $\:{x}_{i}$ is the impact factor of M_R_ containing m features, and $\:{\hat{y}}_{i}$ is the predicted value of M_R_. *k* is the number of the classification and regression tree (CART), *F* is the set of regression trees, and $\:{f}_{k}\left({x}_{i}\right)$ is the predicted value of CART.

For real values $\:{y}_{i}$ the objective function is defined as follows:

2 $$\:\begin{array}{c}Obj=\sum\limits_{i=1}^{n}\:l\left({y}_{i},{\hat{y}}_{i}\right)+\sum\limits_{k=1}^{K}\:\varOmega\:\left({f}_{k}\right),\end{array}$$

where $\:l\left({y}_{i},{\hat{y}}_{i}\right)$ is the loss function used to evaluate the accuracy of $\:{\hat{y}}_{i}$, and $\:{\Omega\:}\left({f}_{k}\right)$ represents the regularization term to avoid overfitting.

Substitute $\:{\hat{y}}_{i}={\sum\limits}_{k=1}^{K}\:{f}_{k}\left({x}_{i}\right)={\hat{y}}_{i}^{(t-1)}+{f}_{t}\left({x}_{i}\right)$ into formula 2, the objective function after *k* iterations is defined as follows:

3 $$\:\begin{array}{c}Ob{j}^{\left(k\right)}=\sum\limits_{i=1}^{n}\:l\left({y}_{i},{\hat{y}}_{i}^{k-1}+{f}_{k}\left({x}_{i}\right)\right)+\varOmega\:\left({f}_{k}\right)+\text{\:}\text{c}\text{o}\text{n}\text{s}\text{t}\text{a}\text{n}\text{t}.\end{array}$$

Use the second-order Taylor expansion of Eq. 3 and delete the constant, the following formula is obtained:

4 $$\:\begin{array}{c}Ob{j}^{\left(k\right)}=\sum\limits_{i=1}^{n}\:\left({g}_{i}{f}_{k}\left({x}_{i}\right)+\frac{1}{2}{h}_{i}{f}_{k}^{2}\left({x}_{i}\right)\right)+\varOmega\:\left({f}_{k}\right),\end{array}$$

where $\:{g}_{i}={l}^{{\prime\:}}\left({y}_{i},{\hat{y}}_{i}^{k-1}\right)$, and $\:{h}_{i}={l}^{{\prime\:}{\prime\:}}\left({y}_{i},{\hat{y}}_{i}^{k-1}\right)$. $\:{g}_{i}$ and $\:{h}_{i}$ represent the first-order and second-order gradient statistics of the loss function respectively.

For a tree containing *L* leaves, which contain *j* sample sets, and the weight of the leaves is $\:\omega\:$, the regularization term $\:{\Omega\:}\left({f}_{k}\right)$ can be expressed as:

5 $$\:\begin{array}{c}\varOmega\:\left({f}_{k}\right)=\lambda\:L+\frac{1}{2}\mu\:\sum\limits_{j=1}^{L}\:{\omega\:}_{j}^{2},\end{array}$$

where λ and µ are constants.

Further simplify the objective function, the following formula is obtained:

6 $$\:\begin{array}{c}Ob{j}^{\left(k\right)}=\sum\limits_{j=1}^{L}\:\left[{G}_{i}{\omega\:}_{j}+\frac{1}{2}\left({H}_{i}+\mu\:\right){\omega\:}_{j}^{2}\right]+\lambda\:L,\end{array}$$

$$\:\text{w}\text{h}\text{e}\text{r}\text{e}\:{G}_{i}={\sum\limits}_{i\in\:{I}_{j}}\:{g}_{i}\:\text{a}\text{n}\text{d}\:{H}_{i}={\sum\:}_{i\in\:{I}_{j}}\:{h}_{i}.$$

### Black-winged kite algorithm

BKA is a biologically inspired algorithm proposed by Wang in 2024^71^. The algorithm replicates black-winged kites’ predation and migration patterns, known for their remarkable ability to adjust to environmental fluctuations in their natural habitat. This simulation strategy enables the method to efficiently adapt to dynamic optimization situations. BKA exhibits an optimal balance between global and local search, a rich population diversity, and simple computational complexity. Furthermore, it demonstrated satisfactory performance in a comparative analysis of 59 test functions with 17 optimization algorithms, including particle swarm optimization, whale optimization algorithm, grey wolf optimization, and sparrow search algorithm^71^. Therefore, BKA is utilized to determine the most suitable settings for XGBOOST, which allows XGBOOST to achieve its highest level of performance. The primary computation of BKA consists of three phases: initialization, predation, and migration. The specifics of this algorithm are as follows:

#### Initialization

As the initial stage of the algorithm, this stage mainly involves the definition of individuals and optimal positions.

Use a matrix to represent the position of black-winged kites.

7 $$\begin{array}{*{20}c} {BK = \left[ {\begin{array}{*{20}c} {BK_{{1,1}} } & {BK_{{1,2}} } & \ldots & \ldots & {BK_{{1,~\dim ~}} } \\ {BK_{{2,1}} } & {BK_{{2,2}} } & \ldots & \ldots & {BK_{{2,~\dim ~}} } \\ \vdots & \vdots & \vdots & \vdots & \vdots \\ \vdots & \vdots & \vdots & \vdots & \vdots \\ {BK_{{pop,1}} } & {BK_{{pop,2}} } & \ldots & \ldots & {BK_{{pop,~\dim ~}} } \\ \end{array} } \right]}, \\ \end{array}$$

where pop is the number of individuals, dim is the dimension of the problem being asked, and *BK*_*i, j*_ represents the *i-th* black-winged kite in the *j-th* dimension.

Use the following formula to represent the position of *i-th* black-winged kite:

8 $$\:\begin{array}{c}{X}_{i}=B{K}_{lb}+{rand}\left(B{K}_{ub}-B{K}_{lb}\right),\end{array}$$

where $\:B{K}_{lb}$ and $\:B{K}_{ub}$ are the lower and upper bounds of the black wing kite in the *j-th* dimension respectively, and rand is a random number between 0 and 1.

Use the root mean square error as the adjustment value and represent the leader ($\:{X}_{\text{L}}$) of the population with the individual with the smallest fitness value, and its position is expressed by the following formula:

9 $$\:\begin{array}{c}{X}_{L}=X\left(find\left({f}_{\text{bett\:}}==f\left({X}_{i}\right)\right)\right),\end{array}$$

where $$\:{f}_{\text{best\:}}=min\left(f\left({X}_{i}\right)\right.$$.

#### Predation

The second stage represents the global exploration and search process of the algorithm by simulating the hunting and searching behavior of black-winged kites on small grassland mammals. The following is the mathematical expression that describes the attacking behavior of black-winged kites.

10 $$\:\begin{array}{c}{y}_{t+1}^{i,j}=\left\{\begin{array}{l}{y}_{t}^{i,j}+n\left(1+\text{sin}\left(r\right)\right)\times\:{y}_{t}^{i,j}p<r\\\:{y}_{t}^{i,j}+n\times\:\left(2r-1\right)\times\:{y}_{t}^{i,j}\:\text{\:else\:}\end{array}\right.,\end{array}$$

11 $$\:\begin{array}{c}n=0.05\times\:{e}^{-2\times\:{\left(\frac{t}{T}\right)}^{2}},\end{array}$$

where $\:{y}_{t}^{ij}$ and $\:{y}_{t+1}^{ij}$ respectively represent the position of the *i-th* black-wing kite in the *j-th* dimension in the *t* and *t + 1* iterations. *r* is a random number between 0 and 1, and *p* is a constant with a value of 0.9. *T* is the maximum number of iterations and *t* is the number of iterations completed so far.

#### Migration

When the climate and food resources are inadequate to support black-winged kites, they will relocate to a different habitat under the direction of a leader in pursuit of more favorable living conditions and increased food availability. In this stage, the leader’s fitness value is utilized to assess their suitability for guiding the populace in migration. If the fitness value of the leader’s current position is lower than that of a randomly selected individual, the leader will relinquish its leadership. In contrast, the leader will guide the populace towards the destination. The subsequent mathematical formula delineates the migratory patterns exhibited by black-winged kites.

12 $$\:\begin{array}{c}{y}_{i+1}^{i,j}=\left\{\begin{array}{cc}{y}_{t}^{i,j}+C\left(\text{0,1}\right)\times\:\left({y}_{t}^{i,j}-{L}_{t}^{j}\right)&\:{F}_{i}<{F}_{ri}\\\:{y}_{t}^{i,j}+C\left(\text{0,1}\right)\times\:\left({L}_{t}^{j}-m\times\:{y}_{t}^{i,j}\right)&\:\:\text{\:else\:}\end{array}\right.,\end{array}$$

13 $$\:\begin{array}{c}m=2\times\:\text{sin}\left(r+\frac{\pi\:}{2}\right),\end{array}$$

where $\:{L}_{t}^{j}$ represents the leader of the black-winged kites in the *j-th* dimension of the *t-th* iteration. $\:{y}_{t}^{i,j}$ and $\:{y}_{i+1}^{i,j}$ respectively represent the position of the *i-th* black-winged kite in the *j-th* dimension in the *t* and *t+1* iterations. $\:{F}_{i}$ represents the position of any black-winged kite in the *j-th* dimension in *t* iteration. $\:{F}_{ri}$ represents the fitness value of a random position in the *j-th* dimension obtained by any black-winged kite in *t* iterations. $\:C\left(\text{0,1}\right)$ represents Cauchy mutation^78^, and its standard formula is $\:f\left(x\right)=\frac{1}{\pi\:}\frac{1}{{x}^{2}+1},\:-{\infty\:}<x<{\infty\:}$.

### Black-winged kite algorithm-extreme gradient boosting

**Fig. 4:**
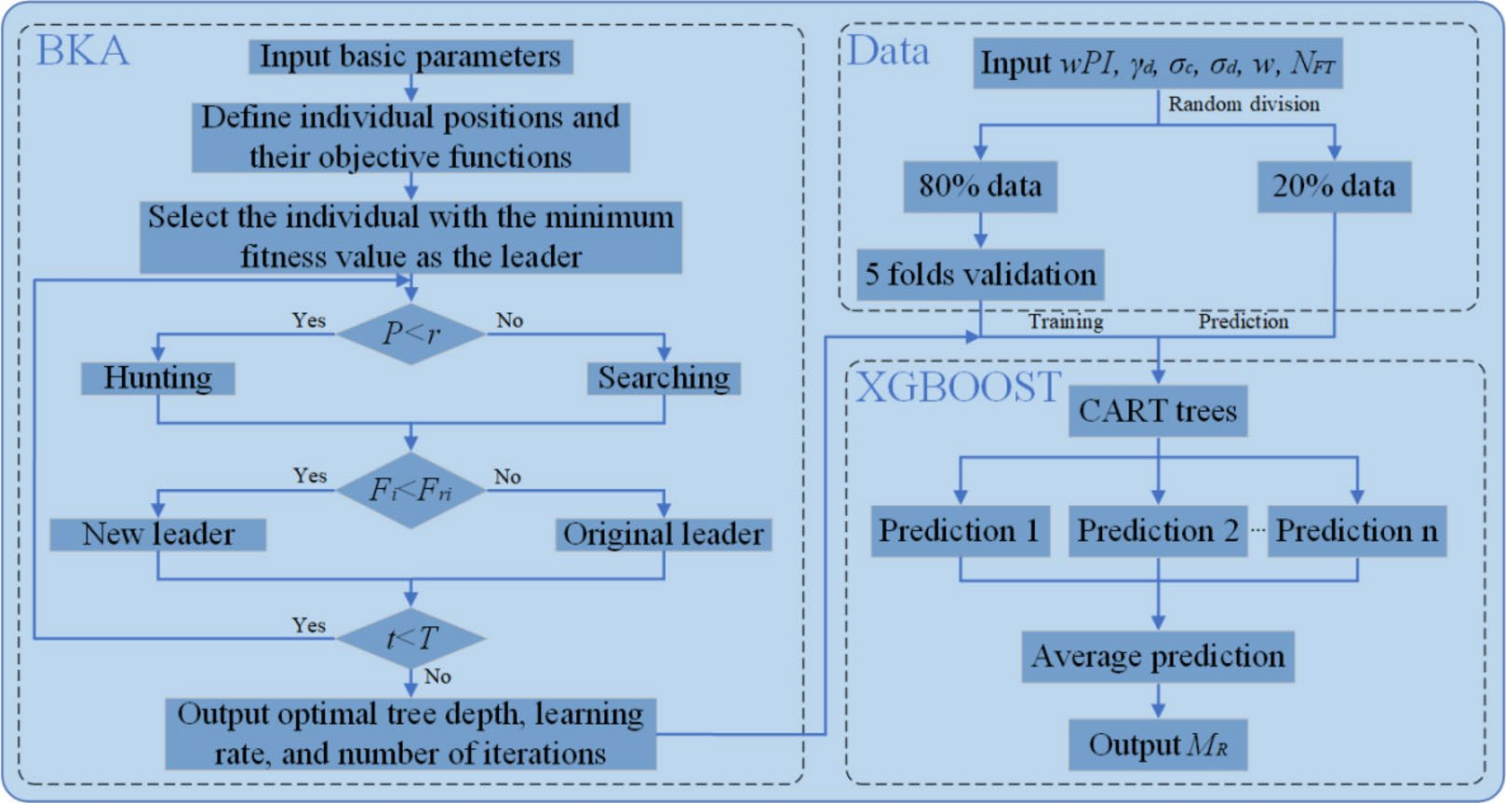
The structure of BKA-XGBOOST.

The BKA-XGBOOST model combines BKA and XGBOOST (Fig. 4). XGBOOST serves as the foundation of the model, processing the input and output data of the soil. Although XGBOOST is proficient in managing nonlinear many-to-one relationships, its performance is most significantly influenced by the depth of the tree, the learning rate, and the maximum number of iterations^79,80^. It is challenging to predict M_R_ using XGBOOST with inappropriate parameter settings accurately^81^. Furthermore, manually identifying optimal parameters necessitates a certain degree of expertise, is also inherently time-consuming, and frequently yields suboptimal outcomes^82^. Consequently, BKA is employed here to ascertain the optimal tree depth, learning rate, and maximum number of iterations for XGBOOST, thereby contributing to the accurate prediction of M_R_. Upon reaching the maximum number of iterations or when the fitness value no longer decreases, BKA will provide an optimal tree depth, learning rate, and maximum number of iterations. Otherwise, BKA will continue to determine the optimal parameters.

Using raw data with disparate magnitudes and sizes along different dimensions for direct training and prediction may result in prolonged training periods and suboptimal training outcomes^83^. Consequently, all the data are transformed using the max-min normalization approach to ensure they fall within the range of 0 to 1. Moreover, the ratio of the training set to the test set significantly influences the predictive performance of XGBOOST. 80% of the data are randomly selected for model training and the remaining data are used for prediction^67,84^. Moreover, a 5-fold cross-validation procedure is employed to mitigate overfitting during training^85,86^. The training data are divided into five distinct subsets in the cross-validation process. One of these subsets is the validation data set, while the other four subsets are used to train the model^68^.

### Performance evaluation

Model performance was assessed using the mean absolute error (MAE), root mean square error (RMSE), determination coefficient (R^2^), mean absolute percentage error (MAPE), and relative percent deviation (RPD). The ideal values of MAE, RMSE, R2, and MAPE are 0, 0, 1, and 0, respectively, and an RPD above 1.4 indicates that the model is reliable^87–90^. The formulas are as follows:

14 $$\:\begin{array}{c}MAE=\frac{1}{n}\sum\limits_{i=1}^{n}\:\left|{y}_{i}-{\hat{y}}_{i}\right|,\end{array}$$

15 $$\:\begin{array}{c}RMSE=\sqrt{\frac{1}{n}\sum\limits_{i=1}^{n}\:{\left({y}_{i}-{\hat{y}}_{i}\right)}^{2}},\end{array}$$

16 $$\:\begin{array}{c}{R}^{2}=1-\frac{\sum\:{\left({y}_{i}-{\hat{y}}_{i}\right)}^{2}}{\sum\:{\left({y}_{i}-\bar{y}\right)}^{2}},\end{array}$$

17 $$\:\begin{array}{*{20}c} {MAPE = \frac{1}{n}\sum\limits_{{i = 1}}^{n} {\left| {\frac{{y_{i} - \hat{y}_{i} }}{{y_{i} }}} \right|} \:,} \\ \end{array}$$

18 $$\:\begin{array}{c}RPD=\frac{SD}{RMSE},\end{array}$$

19 $$\:\begin{array}{c}SD=\sqrt{\frac{1}{n-1}\sum\:_{i=1}^{n}{\left({y}_{i}-\bar{y}\right)}^{2}},\end{array}$$

where $\:{y}_{i}$ represents the actual value, $\:{\hat{y}}_{i}$ represents the estimated value, and $\:\bar{y}$ represents the average of the actual values.

## Results and discussion

### Predictions of the proposed model

The parameter settings of BKA-XGBOOST are primarily determined by the parameters of BKA and the optimization range. The number of populations and the maximum number of iterations of BKA are set to 10 and 20, respectively, to balance the optimization time and effect. The three critical parameters of XGBOOST optimized by BKA are the depth of CART, the learning rate, and the number of iterations of XGBOOST. BKA searches for the optimal parameter within the set range and determines whether the resulting parameter is optimal by the magnitude of the RMSE, which ultimately yields the parameter combination with the smallest RMSE after 20 iterations. The optimization ranges of the three parameters should be set moderately to avoid suboptimal solutions and waste computing resources. Based on previous studies^67,80,91–94^, the optimization ranges of these three parameters are set as follows: the depth of CART is set to 3 to 30, the learning rate is set to 0.001 to 0.1, and the number of iterations of XGBOOST is set to 100 to 1000.

**Fig. 5:**
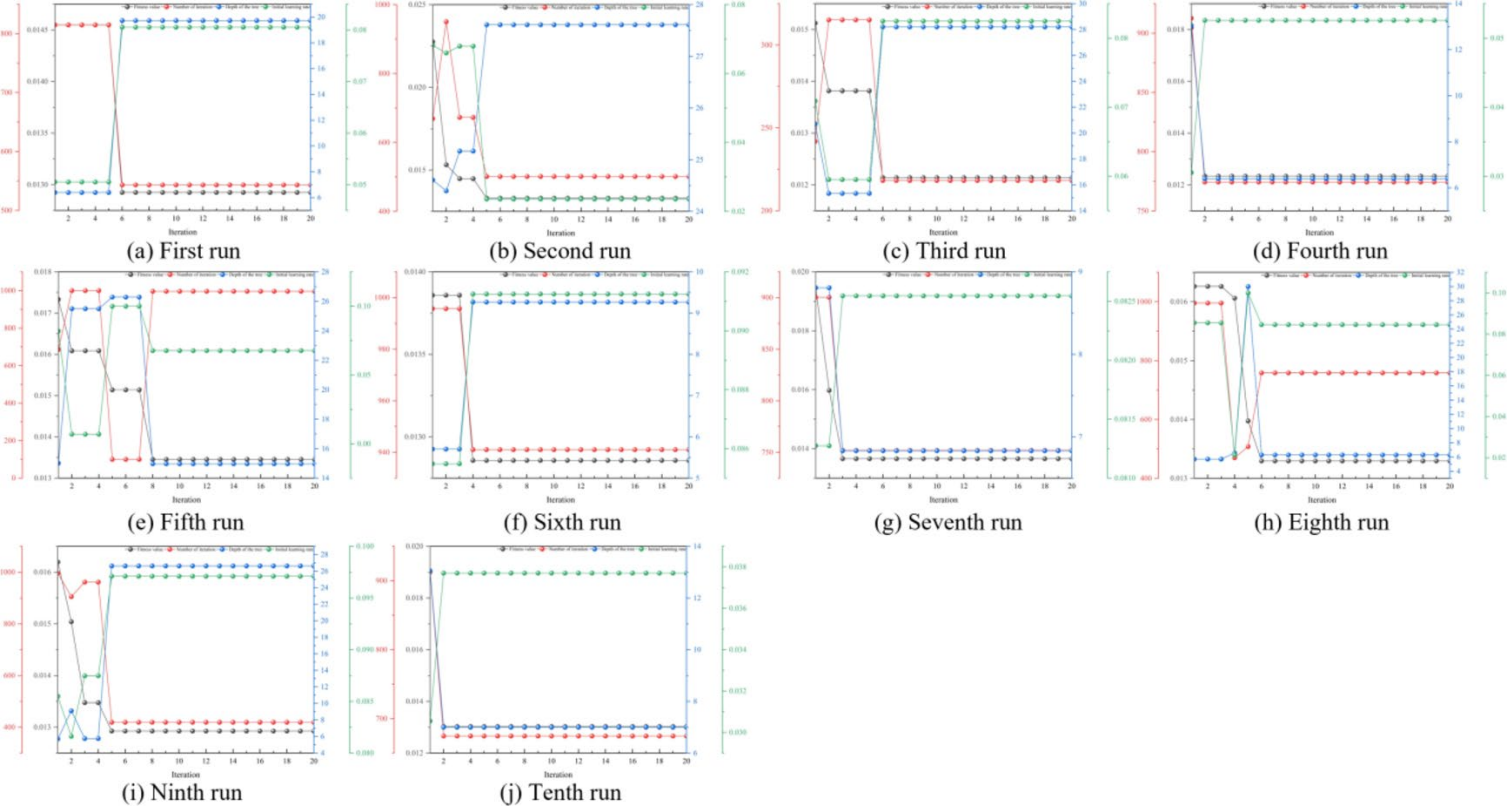
The XGBOOST parameters optimization process.

**Fig. 6:**
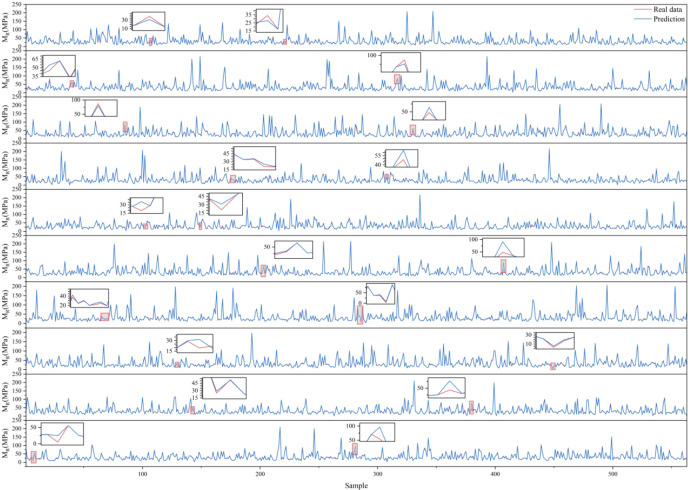
BKA-XGBOOST predictions.

To determine the reliability of BKA-XGBOOST, the original data were randomly and nonrepetitively reassembled ten times. BKA-XGBOOST was employed to predict ten distinct data combinations. The optimization process of BKA and the corresponding optimal parameters are illustrated in Fig. 5; Table 4, respectively. The corresponding parameter combinations obtained from Table 4 indicate that the optimal parameter settings consist of parameter combinations with different values, and there is no apparent linear trend in the parameter settings. Manually determining parameters is a challenging and time-consuming task. Furthermore, the prediction results illustrated in Fig. 6 indicate that the prediction curve aligns well with the actual value curve overall. BKA-XGBOOST, with this parameter configuration, demonstrates great predictive accuracy. This is further substantiated by the indicators shown in Table 5.

**Table 4:** Optimal XGBOOST parameters.

Runs	Iterations	Tree depth	Learning rate
1	543	20	0.0805
2	501	28	0.0236
3	218	28	0.0825
4	774	6	0.0526
5	996	15	0.0677
6	941	9	0.0912
7	752	7	0.0825
8	759	6	0.0846
9	419	27	0.0971
10	675	7	0.0377

**Table 5:** BKA-XGBOOST prediction performance.

Runs	MAE (MPa)	RMSE (MPa)	*R* ^2^	RPD	MAPE
1	0.9126	1.409	0.9968	17.6159	0.0315
2	1.0139	1.6803	0.9965	16.8535	0.0339
3	0.9664	1.6523	0.9965	16.9623	0.0339
4	0.9921	1.7202	0.9954	14.73	0.0347
5	0.9869	1.7138	0.9947	13.7294	0.0382
6	1.028	2.3223	0.9933	12.1773	0.0347
7	0.9065	1.3427	0.9979	21.8586	0.0363
8	0.9605	1.4279	0.997	18.4206	0.0364
9	0.9358	2.0438	0.9927	11.691	0.033
10	1.0471	2.4062	0.9897	9.863	0.0413
Minimum	0.9065	1.3427	0.9897	9.863	0.0315
Maximum	1.0471	2.4062	0.9979	21.8586	0.0413
Average	0.975	1.7719	0.995	15.3902	0.0354

### Sensitivity analysis

**Fig. 7:**
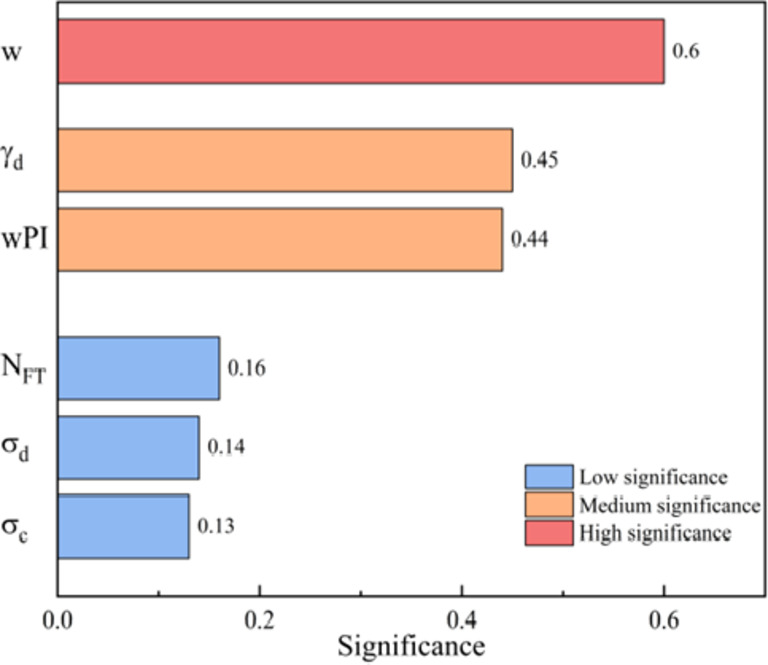
The degree of influence of inputs on M_R_.

Sensitivity analysis aims to determine the relative importance of inputs to outputs. Since the contributions of wPI, γ_d_, σ_c_, σ_d_, w and N_FT_ to the prediction of M_R_ are unknown, the proposed model is still a black box model. Therefore, it is necessary to conduct sensitivity analysis to explore the impact of each variable on M_R_. To further explore the impact of each input on M_R_, the maximum information coefficient (MIC) method was employed. The notion of MIC is derived from mutual information theory and is utilized to quantify the level of mutual effect between two variables^95^. MIC is a method that is universally applicable and provides a fair assessment of the relationships between variables, in contrast to methods such as Pearson, Spearman, and Kendall, which lack these qualities^96–98^. As illustrated in Fig. 7, the influence of w on M_R_ is pronounced, whereas the impacts of N_FT_, σ_d_, and σ_c_ on M_R_ are relatively modest. This suggests that M_R_ depends heavily on w, γ_d_, and wPI.

### Comparison of various models

To ascertain the effectiveness of the proposed model, the models in Table 2 are employed for comparison, including the extreme learning machine (ELM), the K-nearest neighbor (KNN), the support vector machine (SVM), the extreme gradient boosting (XGBOOST), the random forest (RF), the least square support vector machine (LSSVM), the radial basis function neural network (RBFNN), the back propagation neural network (BPNN), and the generalized regression neural network (GRNN). Table 6 presents the parameter settings employed in the comparison models.

**Table 6:** Parameters of comparison models.

Algorithm	Parameter	Value
ELM	Number of nodes in the hidden layer	400
KNN	Number of nearest neighbors	4
SVM	Regularization parameter	50
	Kernel function parameter	10
LSSVM	Regularization parameter	0.1
	Kernel function parameter	100
XGBOOST	Tree depth	15
	Learning rate	0.01
	Maximum iterations	400
RF	Trees	20
	Tree depth	5
RBFNN	Spreading rate	5
BPNN	Number of nodes in the hidden layer	7
	Learning rate	0.01
GRNN	Spreading rate	0.01

**Fig. 8:**
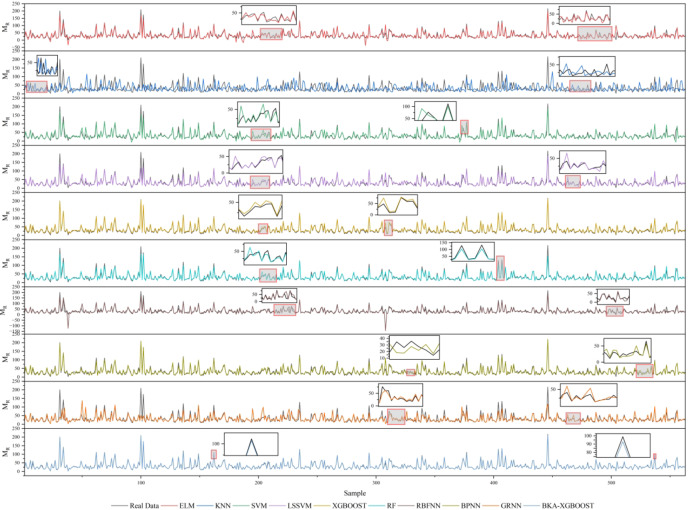
Predictions from different models.

**Fig. 9:**
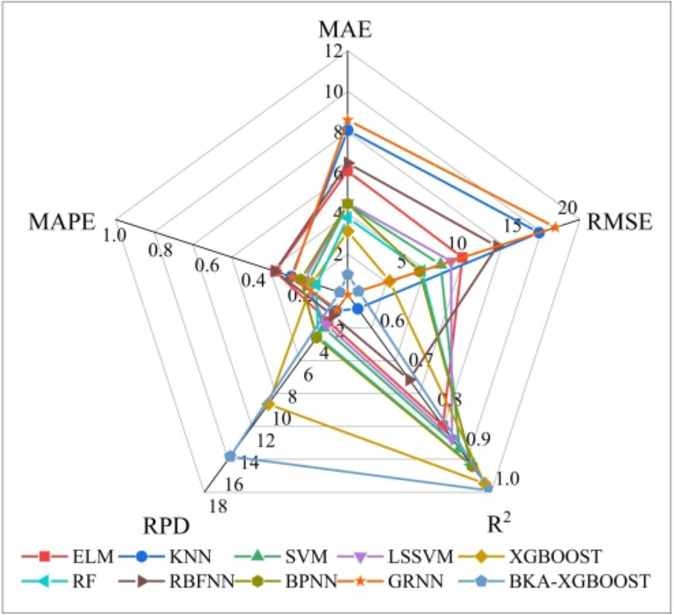
Performance of various models.

The dataset used for the comparison model is the 4th dataset in Table 4 (BKA-XGBOOST’s performance on this dataset is closest to its average performance, and the feasibility of the proposed model can be fairly evaluated by comparing its performance on this dataset). Figure 8 shows the predictions of different models for the same dataset. According to Fig. 8, the prediction curve of KNN is far from the actual value curve, and the absolute prediction error for most samples is the largest. On the other hand, the prediction curve of BKA-XGBOOST is closer to the actual value, and the error is the smallest in all samples except the 407th sample. Based on the model performance comparison shown in Fig. 9, XGBOOST outperforms the other single models, indicating that using XGBOOST to predict M_R_ is reasonable. In addition, the performance of XGBOOST optimized by BKA has improved. The proposed model accomplishes the objective of precisely forecasting M_R_.

### Score analysis

Score analyze is a method for evaluating model performance^99,100^. In score analysis, models are allocated scores based on various evaluation metrics, and the model with the performance metrics closest to the desired values requires the highest score^101–103^. This study evaluates ten models from five error metrics with a maximum score of 10 and a minimum score of 1. The scores obtained by the models are presented in Table 7. From Table 7, it can be seen that BKA-XGBOOST has received the highest score and is the best-performing model. XGBOOST follows.

**Table 7:** The models’ scores.

Model	MAE	RMSE	*R* ^2^	RPD	MAPE	Total
ELM	4	4	4	4	2	18
KNN	2	2	2	2	3	11
SVM	7	6	6	6	7	32
LSSVM	5	5	5	5	6	26
XGBOOST	9	9	9	9	8	44
RF	8	7	7	7	9	38
RBFNN	3	3	3	3	1	13
BPNN	6	8	8	8	5	35
GRNN	1	1	1	1	4	8
BKA-XGOOST	10	10	10	10	10	50

### Regression error characteristics curve

**Fig. 10:**
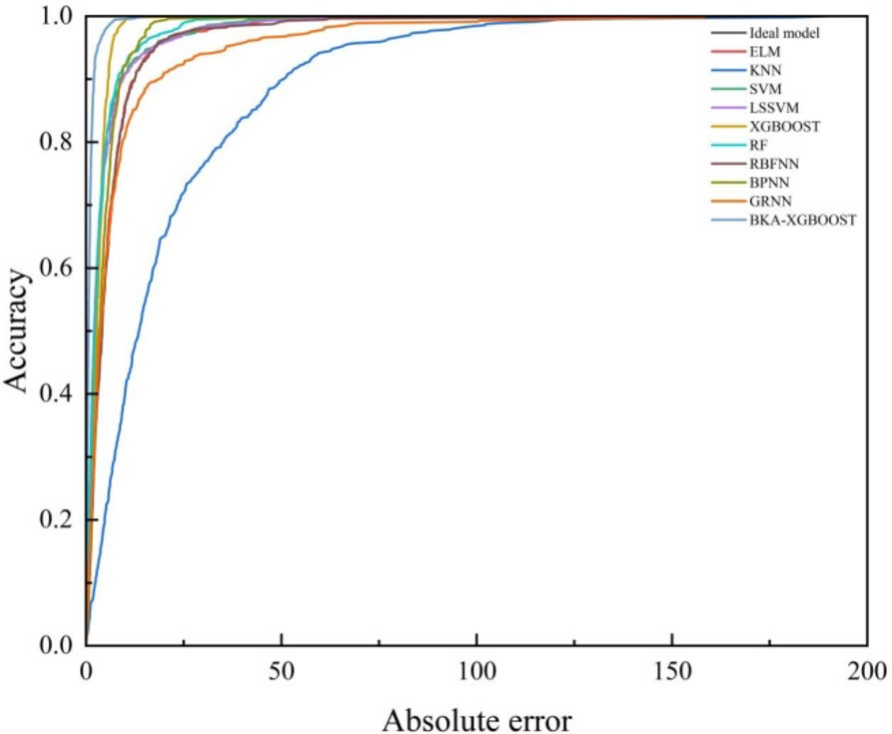
The models’ REC curves.

Regression error characteristic (REC) curve visually depicts a regression model’s predictive performance. REC curve contrasts with conventional merits by emphasizing the cumulative distribution function of the absolute error^104^. The area over the REC curve (AOC) represents a biased estimate of the predicted error^105^, with the lowest AOC value signifying the most effective model^106^. To evaluate the models’ performance, the REC curves were generated (Fig. 10) and the AOC was computed (Table 8). Table 8 demonstrates that the AOC of BKA-XGBOOST is the lowest, signifying it as the most effective model.

**Table 8:** The models’ AOC.

Model	AOC	Model	AOC
ELM	91.6282	RF	57.7266
KNN	169.0302	RBFNN	186.3321
SVM	65.7152	BPNN	57.15
LSSVM	88.8714	GRNN	150.3044
XGBOOST	22.7709	BKA-XGBOOST	14.3497

### Anderson–Darling test

The Anderson-Darling (AD) test is a nonparametric test typically used to assess data’s normality and analyze the dispersion of predictions. If the model’s AD value is close to the AD value of the actual value, the model demonstrates a reliable capacity to evaluate the M_R_^107^. The AD test was employed to evaluate the fit of the models’ predictions to the actual value. As shown in Table 9, the P of the actual value and all predictions are below 0.005, indicating that they are non-normally distributed. Moreover, the AD value of BKA-XGBOOST is closest to the AD value of the actual value, which indicates that BKA-XGBOOST has the best performance.

**Table 9:** Anderson-Darling test results.

Models	Mean	StDev	AD	*P*
Actual	32.583	25.305	42.288	< 0.005
ELM	32.301	24.493	26.561	< 0.005
KNN	31.9	19.313	32.303	< 0.005
SVM	32.811	23.463	33.95	< 0.005
LSSVM	32.723	22.134	26.468	< 0.005
XGBOOST	35.395	24.666	45.176	< 0.005
RF	32.8	21.969	39.711	< 0.005
RBFNN	31.884	25.341	30.811	< 0.005
BPNN	32.728	24.609	43.11	< 0.005
GRNN	31.594	19.664	33.807	< 0.005
BKA-XGBOOST	32.698	25.205	42.056	< 0.005

### Comparison models in the literature

Table 10 shows the comparison with previous studies using different models, including genetic algorithm-adaptive layered population structure (GA-ALPS), symbiotic organisms search-least square support vector machine (SOS-LSSVM), jellyfish swarm optimizer- extreme gradient boosting (JSO-XGBOOST) and genetic algorithm-artificial neural network (GA-ANN). As the previous research utilizes a different dataset than the one employed in this work, direct performance comparisons are unconvincing. The proposed model retains the same parameters as previously stated and predicts the dataset, including 283 data points utilized by Ghorbani (Additional data 2), and 891 data points employed by Sadik, Azam, and He (Additional data 1). According to Table 10, the proposed model demonstrated the highest level of accuracy in predicting outcomes. The RMSE of the proposed model is reduced by 13.72% and 24.82% compared to GA-ANN and JSO-XGBOOST, respectively. The proposed model improves the prediction accuracy to a certain extent, and this research contributes to the accurate prediction of M_R_.

**Table 10:** Comparison with previous studies.

Authors	Model	Number of data	MAE (MPa)	RMSE (MPa)	*R* ^2^
Sadik^61^	GA-ALPS	891 (additional data 1)	5.72	7.62	0.93
Azam^62^	SOS-LSSVM	891 (additional data 1)	4.399	6.724	0.942
He^63^	JSO-XGBOOST	891 (additional data 1)	–	5.489	0.965
Ghorbani^65^	GA-ANN	283 (additional data 2)	4.02	5.2	0.97
This study	BKA-XGBOOST	283 (additional data 2)	3.1306	4.4862	0.9816
		891 (additional data 1)	2.8754	4.1266	0.9769
		2813 (experimental data)	0.975	1.7719	0.995

## Model programing

**Fig. 11:**
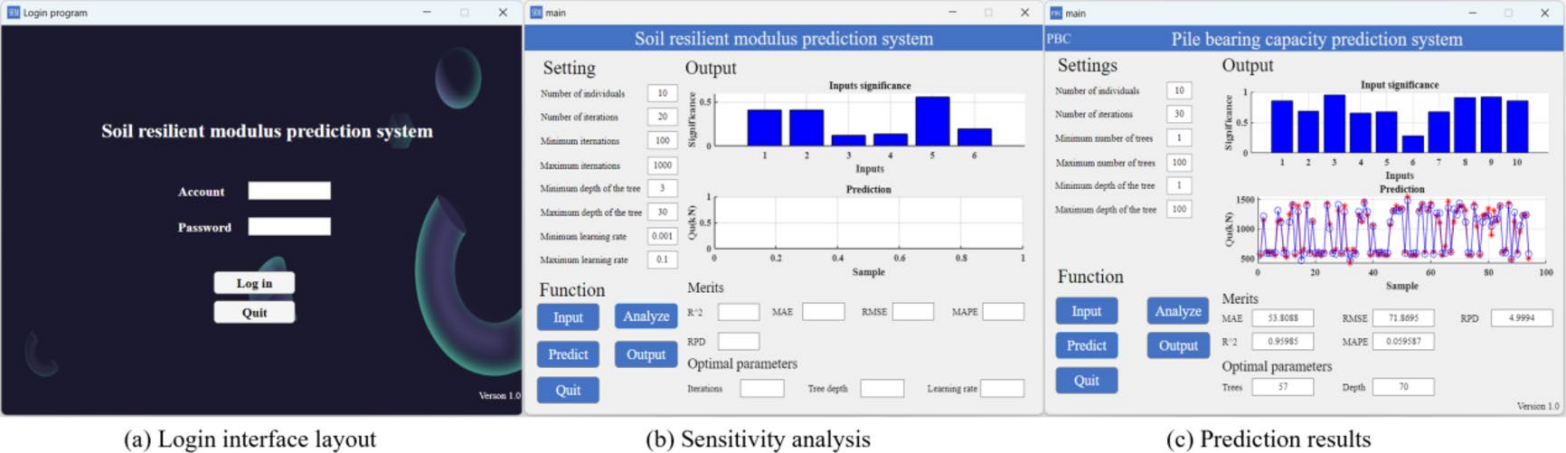
Soil resilient modulus prediction program.

To increase the practicality of the model, MATLAB App Designer is employed to create the M_R_ prediction program. The program is divided into two interfaces: the login interface and the main program (illustrated in Fig. 11). Prior to accessing the main program, users are required to enter their accounts and passwords on the login interface. Upon successful verification, users are permitted to enter the main program. The main program is divided into three sections: the setting area, the functional area, and the output area. The setting area reserves the default BKA basic parameters and the optimization range of XGBOOST parameters, which users have the option to adjust. The ribbon contains Input, Analyze, Predict, Output, and Quit buttons. By clicking the Input button, the data are imported into the program, which supports files in the formats xls, csv, and xlsx. If the user wants to explore the sensitivity of different input factors to M_R_, the importance of the inputs calculated by the MIC method (the inputs are represented by numbers in order) will be shown by clicking the Analyze button. Subsequently, clicking the Predict button initiates the random selection of 80% of the data for training, with the objective of identifying the optimal XGBOOST parameters. The remaining data are then predicted on the basis of the obtained parameters. The prediction performance of the model is displayed in the form of a line chart, and the prediction accuracy of the model is evaluated with five evaluation indicators. In addition, the program also displays the optimal number of iterations, tree depth, and learning rate obtained through BKA. After clicking the Output button to obtain the prediction results, the user can click the Quit button to exit the program.

## Summary and conclusions

Forecasting M_R_ is an important part of assessing road safety and sustainability. Traditional methods, including experimental, regression equation, and constitutive model methods, are difficult to implement in engineering due to their time-consuming and complex characteristics. The generality and accuracy of some machine learning methods for predicting M_R_ also need to be further improved. In this situation, this study proposes BKA-XGBOOST as a novel approach for predicting M_R_. The performance of the proposed model is compared with nine individual models and four literature models using several metrics. The conclusions drawn from this study are as follows:

- Influence of geotechnical factors: Among the six geotechnical factors utilized as inputs, the one that has the most impact on M_R_ is w, which is influenced by γ_d_.
- Effect of optimization algorithm: For different combinations of soil data, BKA can provide the optimal parameters to XGBOOST to ensure its outstanding performance. Moreover, the AOC of XGBOOST following BKA optimization is reduced significantly and further improved in accuracy.
- The best performance model: Performance metrics (MAE = 0.975 MPa, RMSE = 1.7719 MPa, R^2^ = 0.995, RPD = 15.3902, MAPE = 0.0354), score analysis (score = 50), REC curve (AOC = 14.3497), AD test (model’s AD = 42.056, a difference of 0.232 from the AD of the actual value) indicate that BKA-XGBOOST is the best model. It also proved to be the best model in comparison with four literature models (MAE = 2.8754 MPa, RMSE = 4.1266 MPa, R^2^ = 0.9769 for additional dataset 1; MAE = 3.1306 MPa, RMSE = 4.4862 MPa, R2 = 0.9816 for additional dataset 2).

In conclusion, this study introduced a new model, BKA-XGBOOST, for evaluating MR. The proposed model exhibits great accuracy, generalizability, and user-friendliness. It has potential for application in geotechnical engineering. However, the soil types employed in the experiments are inadequate. Consequently, more types of soil data will be used in subsequent experiments to test the model’s generalizability and precision further.

## Data Availability

All data generated or analyzed during this study are included in this manuscript.

## Abbreviations

AD: Anderson-Darling
AOC: Area over the REC curve
BKA: Black black-winged kite algorithm
BPNN: Back propagation neural network
ELM: Extreme learning machine
GA-ALPS: Genetic algorithm-adaptive layered population structure
GA-ANN: Genetic algorithm-artificial neural network
GBDT: Gradient boosting decision tree
GRNN: Generalized regression neural network
JSO-XGBOOST: Jellyfish swarm optimizer- extreme gradient boosting
KNN: K-nearest neighbor
LSSVM: Least square support vector machine
MAE: Mean absolute error
MAPE: Mean absolute percentage error
MIC: Maximum information coefficient
M_R_: Soil resilient modulus
N_FT_: Number of FT cycles
R^2^: Determination coefficient
RBFNN: Radial basis function neural network
REC: Regression error characteristics curve
RF: Random forest
RMSE: Root mean square error
RPD: Relative percent deviation
SOS-LSSVM: Symbiotic organisms search-least square support vector machine
SVM: Support vector machine
w: Moisture content
wPI: Weighted plasticity index
XGBOOST: Extreme gradient boosting
γ_d_: Dry unit weight
ε_r_: Recoverable strain
σ_c_: Confining stress
σ_d_: Ratio of deviator stress
